# Electric Double Layer and Orientational Ordering of Water Dipoles in Narrow Channels within a Modified Langevin Poisson-Boltzmann Model

**DOI:** 10.3390/e22091054

**Published:** 2020-09-21

**Authors:** Mitja Drab, Ekaterina Gongadze, Veronika Kralj-Iglič, Aleš Iglič

**Affiliations:** 1 Faculty of Electrical Engineering, Tržaška Cesta 25, University of Ljubljana, SI-1000 Ljubljana, Slovenia; mitja.drab@fe.uni-lj.si (M.D.); ekaterina.gongadze@fe.uni-lj.si (E.G.); 2 Faculty of Health Sciences, Zdravstvena Pot 5, University of Ljubljana, SI-1000 Ljubljana, Slovenia; kraljiglic@gmail.com

**Keywords:** electric double layer, orientational ordering of water dipoles, Helmholtz free energy, modified Langevin Poisson-Boltzmann model

## Abstract

The electric double layer (EDL) is an important phenomenon that arises in systems where a charged surface comes into contact with an electrolyte solution. In this work we describe the generalization of classic Poisson-Boltzmann (PB) theory for point-like ions by taking into account orientational ordering of water molecules. The modified Langevin Poisson-Boltzmann (LPB) model of EDL is derived by minimizing the corresponding Helmholtz free energy functional, which includes also orientational entropy contribution of water dipoles. The formation of EDL is important in many artificial and biological systems bound by a cylindrical geometry. We therefore numerically solve the modified LPB equation in cylindrical coordinates, determining the spatial dependencies of electric potential, relative permittivity and average orientations of water dipoles within charged tubes of different radii. Results show that for tubes of a large radius, macroscopic (net) volume charge density of coions and counterions is zero at the geometrical axis. This is attributed to effective electrolyte charge screening in the vicinity of the inner charged surface of the tube. For tubes of small radii, the screening region extends into the whole inner space of the tube, leading to non-zero net volume charge density and non-zero orientational ordering of water dipoles near the axis.

## 1. Introduction

The electric double layer (EDL) is a central phenomenon found at the boundary between a charged surface and an electrolyte solution [1,2,3,4,5,6,7,8]. The counterions are accumulated close to the charged surface and the coions are depleted from this region, resulting in a non-homogeneous distribution of ions. The physical properties of the EDL are crucial in understanding colloidal systems, transport of charged molecules across biological membrane channels or binding of charged proteins to biological surfaces.

Recently, much attention is being devoted to inorganic and organic hollow cylindrical structures in the nanometer range due to their potential benefit in technology, biology and medicine [9]. Potential applications range from microelectronics to microfluidics [10]. Ion channels or pores in biological membranes and blood capillaries are also examples for cylindrical nanotubes.

In some biological systems, the walls of organic nanotubes are charged and in contact with electrolyte solution, where the primary agents of interaction are electrostatic forces, both between charged particles and polar water molecules. Due to the surface charge of the walls, counterions and coions of the electrolyte are, respectively, accumulated and depleted near the walls. At the internal surfaces concave electrical double layers of cylindrical geometry are formed [11].

Furthermore, when bound to a cylindrical geometry, the effect of curvature on EDL properties is significant on small enough scales. Such biological cylindrical channels, where EDL interactions are important, encompass axons or tunneling nanotubes [12]. When artificially made channels, for example, those found in nanoporous materials, are used in the manufacture of electrochemical nanocapacitors, their power and energy densities are dependent on EDL characteristics such as capacitance [13,14,15].

EDL was first modeled by Helmholtz who assumed that the charged surface attracts the surrounding point-like counterions and a single layer is formed to screen the charge [16,17]. Later, these ions have been described by a Boltzmann distribution, forming a diffuse layer extending into the bulk [18,19]. The finite size has been incorporated by Stern with the so-called distance of closest approach [20] and later developed further by numerous authors [3,21,22,23,24,25,26]. In recent decades, EDL has been the subject of numerous analytical and numerical studies from Monte-Carlo methods, DFT theories and lattice models [3,7,27,28,29,30,31,32,33,34,35,36,37,38,39,40,41,42,43,44]. Additionally, interest in nanostructured materials [45,46,47,48] requires that theoretical models of EDL are revisited [49,50,51], also by taking into account the possible quantum effects [52,53].

It has been shown that close to the charged surface, orientational ordering and depletion of water molecules may result in a strong decrease in the local permittivity of the electrolyte solution [54,55,56,57,58,59,60,61]. Considering the orientational ordering of water and finite size of molecules, Outhwaite and collaborators developed a modified Poisson-Boltzmann’s (PB) theory of EDL composed of a mixture of hard spheres with point-like dipoles and finite-sized ions [54,62]. Later, Szalai et al. [63] published a mean spherical approximation-based theory [64] that can reproduce simulation results for the electric field dependence of the dielectric permittivity of a dipolar fluid in a saturation regime. The problem was also considered within a discrete lattice statistics model taking into account the asymmetric size of ions and orientational ordering of water dipoles [44]. Recently, ion-ion and ion-water correlations were also considered in a mean-field approach [65,66].

**Figure 1:**
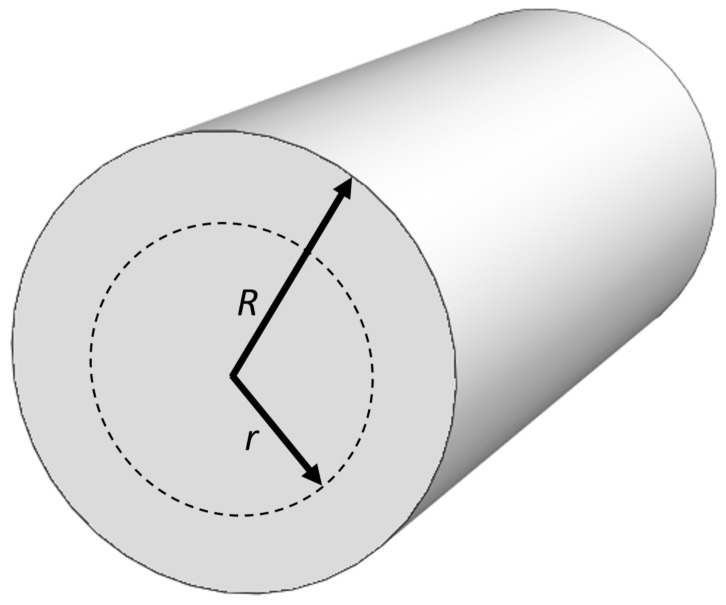
A schematic of a tubular structure with labeled independent coordinate *r* that can be at most *R*.

In the present paper, we first discuss the relative permittivity of water molecules within a cavity field model. We then go on to the derivation of a modified Langevin Poisson-Boltzmann (LPB) equation for point-like ions and water dipoles for planar geometry and then generalize the equations for arbitrary geometry. In derivation of modified LPB equation we construct a Helmholtz free energy functional and minimize it to derive the analytical expressions for ion distributions and spatial dependence of statistical averages orientations of water dipoles. The free energy expression also includes contributions from configurational entropy of ions and rotational entropy of water dipoles. In the second part of the paper the modified LPB equation and the corresponding boundary conditions, generalized for an arbitrary geometry, are utilized to present the numerical solution for a cylindrical geometry with special emphasis given to very narrow cylindrical channels (Figure 1).

## 2. Relative Permittivity of Water

The dipole moment of an isolated water molecule is around 1.85 D (Debye is $3.336\times 10^{-30}$ Cm). In a solution, the dipole moment of a single water molecule differs from an isolated one since each molecule is also polarized by the electric field of the neighboring water molecules, creating an effective value of the dipole moment around 2.4 D–2.6 D [67,68]. The body of literature dealing with the dielectric permittivity of water is voluminous and comprehensive, from analytic models detailing the state of bound water molecules and water in charged crevices [69,70] to molecular dynamics simulations with nonlinear response to external electric fields [71,72].

The effect of a polarizing environment can be reproduced in the most simple way by introduction of the cavity field [61,73,74,75]. Cavity field is derived by solving the Poisson’s equation of a model water molecule placed in an outside homogeneous electric field (for a detailed derivation, see Reference [76]). The present section deals with polarization of water dipoles that follows directly from the cavity field.

**Figure 2:**
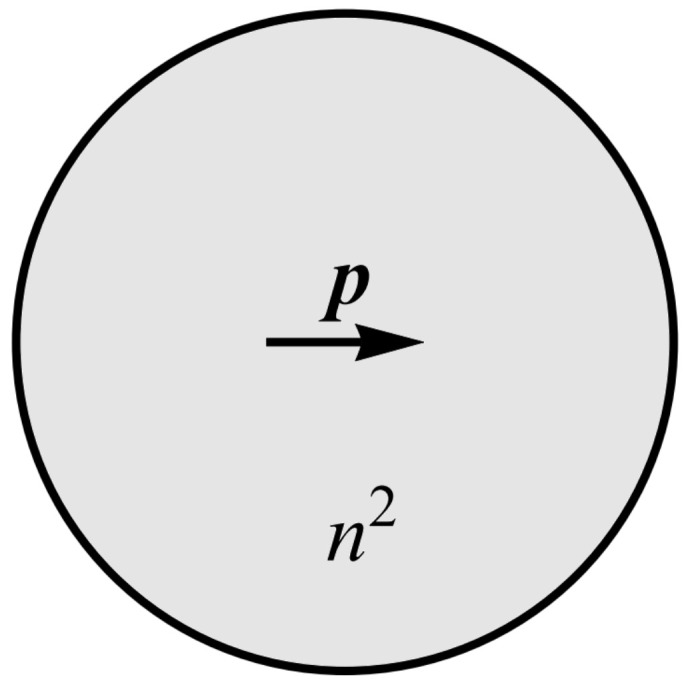
A single water molecule is modelled by a sphere with relative permittivity $n^{2}$, where n=1.33 is the refractive index of water. A permanent point-like rigid dipole with magnitude, *p*, is located at the center of the sphere [61]. Due to the built up charge, the point dipole experiences the so-called cavity field $E_{c}$.

The water molecules are described within the modified Kirkwood approach [75] as point-like dipoles p with magnitude |p|=p at the centres of finite sized spheres, embedded in a medium with electric permittivity representing the ion-water solution $\varepsilon_{r}$ (Figure 2) [7,61]. Within this medium, a spatially homogeneous electric field, E, is present. Due to the built up charge at the interface between the inside and outside of the sphere, the dipole experiences the so called cavity field $E_{c}$. The relative permittivity of water is given by $\varepsilon_{r}=1+P_{\mathrm{tot}}/\left(\varepsilon_{0}E\right)$, where $P_{\mathrm{tot}}$ is the total polarization of water dipoles, *E* is the magnitude of the spatially homogeneous electric field and $\varepsilon_{0}$ is the permittivity of vacuum. The total polarization is the sum of electronic polarization, $P_{e}$, and orientational polarization due to the permanent water dipoles *P*, so that $P_{\mathrm{tot}}=P_{e}+P$. The electronic polarization determines the refractive index of water [51,61] $n^{2}=1+P_{e}/\left(\varepsilon_{0}E\right)\approx 1.8$ and $\varepsilon_{r}$ can be expressed as

(1) $$\varepsilon_{r}=n^{2}+\frac{P}{\varepsilon_{0}E}.$$

To find the expression for *P* we must take into account the constant number density of water $n_{w}$ and the statistical-average orientation of water molecules in the solution [7]:

(2) $$P=n_{w}p\langle \cos\theta \rangle .$$

**Figure 3:**
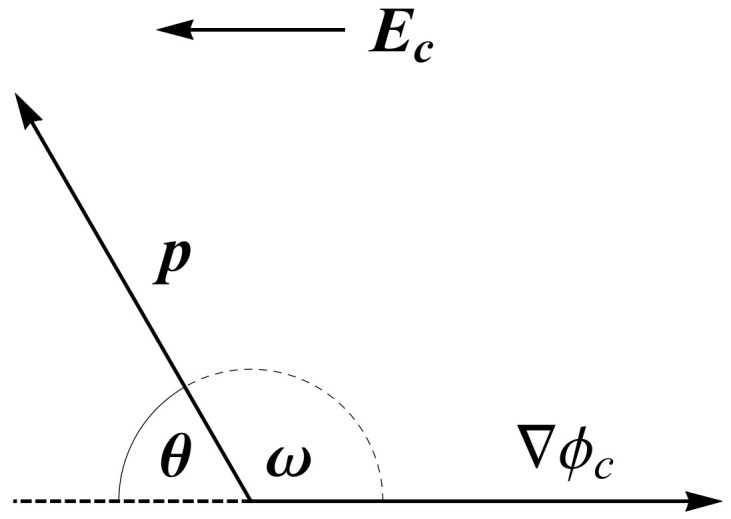
Relation between angles θ and ω. The water internal dipole moment is marked by p, the local cavity field, $E_{c}$, points in the opposite direction of $\nabla \phi_{c}$.

Here, θ is the angle between p and the cavity field $E_{c}$ acting on it (see Figure 3). Statistical averaging is labeled by 〈…..〉. To estimate 〈cosθ〉, we must first find the expression for $E_{c}$. This involves solving the Poisson equation for a sphere with electric permittivity $n^{2}$ embedded in a medium with a relative permittivity $\varepsilon_{r}$ described in detail in Reference [76]. Neglecting the short range interactions between dipoles, the local electric field strength at the centre of the sphere at the location of the permanent point-like dipole (Figure 2) can be expressed as [7,76]

(3) $$E_{c}=\frac{3\varepsilon_{r}}{n^{2}+2\varepsilon_{r}}E.$$

When the surrounding medium has a relative permittivity much larger than the refractive index of water $\varepsilon_{r}\gg n^{2}$, it follows that

(4) $$E_{c}\approx \frac{3}{2}E\;\to \;E_{c}\approx \frac{3}{2}E.$$

So far we have neglected the reaction field, which is the field of the point-like dipole at the center of the cavity itself. This reaction field is directly proportional to the strength of dipole $E_{\mathrm{react}}\propto p$. In vacuum, in the case of a single isolated water molecule, the external dipole moment is also the experimentally measured dipole moment of a single water molecule $p_{e}$ given by [7,76]:

(5) $$p_{e}=\frac{3}{n^{2}+2}\;p.$$

Here, p is the permanent point-like internal water dipole at the center of the sphere. The energy of an internal point-like dipole p in a local field $E_{c}$ is [61]

(6) $$W_{e}=-p\cdot E_{c}.$$

Substituting from Equation (4) and Equation (5), we can express the dipole energy as [61]

(7) $$\begin{array}{ccc} W_{e} & = & -\frac{3}{2}\left(\frac{2+n^{2}}{3}\right)p_{0}E\cos\theta , \end{array}$$

(8) $$\begin{array}{ccc} W_{e} & = & \gamma p_{0}E\cos\omega . \end{array}$$

Here, $p_{0}$ is the magnitude of $p_{e}$ and ω is supplementary to θ, as shown in Figure 3. The constant γ equals [7,61] (see Equations (7) and (8))

(9) $$\gamma =\frac{3}{2}\left(\frac{2+n^{2}}{3}\right).$$

With this in mind, the ensemble average in Equation (2) can be calculated as:

(10) $$\langle \cos\omega \rangle =\frac{\int \cos\omega e^{-(\beta \gamma p_{0}E\cos\omega )}\;d\Omega }{\int e^{-(\beta \gamma p_{0}E\cos\omega )}\;d\Omega }=-L\left(\beta \gamma p_{0}E\right).$$

Here, β is the Boltzmann’s factor equal to β=1/kT, where kT is the thermal energy. The element of solid angle is dΩ=2πsinωdω, meaning that the integral runs from 0 to π with assumed azimuthal symmetry. The Langevin function is defined as L(u)=cothu−1/u. By taking into account Equations (1), (2), (5) and (10), we can express the relative water permittivity as [7]:

(11) $$\varepsilon_{r}=n^{2}+\frac{n_{w}p_{0}}{\varepsilon_{0}}\left(\frac{2+n^{2}}{3}\right)\frac{L(\beta \gamma p_{0}E)}{E}.$$

In the limit of vanishing electric field strength (E→0), the above expression for the relative permittivity of water yields to the Onsager limit [7]

(12) $$\varepsilon_{r}=n^{2}+\frac{n_{w}p_{0}^{2}\beta }{2\varepsilon_{0}}{\left(\frac{2+n^{2}}{3}\right)}^{2}.$$

For $p_{0}=3.1$ D and $n_{w}/N_{A}=55$ mol/L, [7,44], where $N_{A}$ is the Avogadro number, Equation (12) yields the value $\varepsilon_{r}=78.5$ at room temperature. Returning to Equation (2), we can write the final result for the orientational polarization of water dipoles *P*, which will be needed for our Helmholtz free energy minimization in the following section:

(13) $$P=-n_{w}p_{0}\left(\frac{2+n^{2}}{3}\right)L\left(\beta \gamma p_{0}E\right).$$

## 3. Derivation of the Modified LPB Equation by Minimization of Helmholtz Free Energy

Our model assumes the electrolyte solution is a mixture of point-like monovalent co- and counterions and permanent water dipoles, representing the water molecules. The expression for the spatial dependence of the solution permittivity $\varepsilon_{r}\left(x\right)$, arising as a direct consequence of the spontaneous ordering of water dipoles, can be obtained by using the minimization of the Helmholtz free energy in a one-dimensional setting with the charged planar surface located at x=0. In the minimization procedure, the local electric field at the positions of the hydrated point-like ions in the electrolyte solution is denoted by E(x), while the local cavity field at the positions of the water internal point-like dipoles is denoted by $E_{c}\left(x\right)$. We can write the Helmholtz free energy of the system *F* as (see also Reference [58]):

(14) $$\begin{array}{cc} F & =\underset{F_{1}}{\underset{︸}{\frac{\varepsilon_{0}n^{2}}{2}\int E_{c}^{2}\left(x\right)\;dV}}+\underset{F_{2}}{\underset{︸}{\frac{\varepsilon_{0}n^{2}}{2}\int E^{2}\left(x\right)\;dV}}\;+kT[\underset{F_{3}}{\underset{︸}{\int (n_{+}\left(x\right)\ln\frac{n_{+}\left(x\right)}{n_{0}}-\left(n_{+}\left(x\right)-n_{0}\right))\;dV}}\;+\\ \; & +\underset{F_{4}}{\underset{︸}{\int \left(n_{-}\left(x\right)\ln\frac{n_{-}\left(x\right)}{n_{0}}-\left(n_{-}\left(x\right)-n_{0}\right)\right)\;dV}}\;+\underset{F_{5}}{\underset{︸}{\int (\lambda_{+}n_{+}\left(x\right)+\lambda_{-}n_{-}\left(x\right))\;dV}}\;+\\ \; & +\underset{F_{6}}{\underset{︸}{\int n_{w}{\langle P\left(x,\omega \right)\ln P(x,\omega )\rangle }_{\omega }\;dV}}\;+\underset{F_{7}}{\underset{︸}{\int n_{w}\eta \left(x\right)\left({\langle P\left(x,\omega \right)\rangle }_{\omega }-1\right)\;dV}}]. \end{array}$$

The thermal energy is given by kT, while *n* is the refractive index. For greater clarity, we split the particular contributions to the free energy as marked by the underbraces in Equation (14). The mean field created by coions and counterions and the water dipoles polarization contribution are given by terms $F_{1}$ and $F_{2}$, respectively. Mixing entropy free energy contributions of point-like counterions and coions are accounted for in terms $F_{3}$ and $F_{4}$. The constraint of a constant number of ions in the system is given in $F_{5}$, where $\lambda_{+}$ and $\lambda_{-}$ are the global Lagrange’s multipliers for counterions and coions. The free energy that corresponds to orientational entropy of permanent water dipoles is given by $F_{6}$, while the last term, $F_{7}$, gives the local constraint for orientation of dipoles. P(x,ω) is the probability that a permanent water dipole located at *x* is oriented at angle ω with respect to the normal to the charged surface (Figure 3). The brackets ${\langle \ldots \rangle }_{\omega }$ denote the average:

(15) $${\langle F\left(x,\omega \right)\rangle }_{\omega }=\frac{1}{4\pi }\int_{0}^{\pi }F\left(x,\omega \right)\;2\pi \sin\omega d\omega .$$

Here, ω is the angle between the internal dipole moment vector, p, and $n_{\phi }=\nabla \phi_{c}/\left|\nabla \phi_{c}\right|$ (see Figure 3). We perform variation on the Helmholtz free energy, *F*, in Equation (14), so that δF=0. Let us deal with the variational approach of every contribution in Equation (14) particularly, beginning with $F_{1}$ and $F_{2}$. For clarity of notation, direct spatial dependence will sometimes be omitted, so that for example, $n_{+}\left(x\right)\equiv n_{+}$.

### 3.1. Variation Procedure

#### 3.1.1. Electric Fields ($F_{1}$ and $F_{2}$)

Since there are no time dependent magnetic fields, we can express the electric fields as potentials $E\left(x\right)=-\phi^{\prime }\left(x\right)$, $E_{c}\left(x\right)=-\phi_{c}^{\prime }\left(x\right)$, where the prime labels the spatial derivative, and perform a variation on the electrostatic term pertaining to water dipoles.

(16) $$\delta \left(\frac{\varepsilon_{0}n^{2}}{2}\int \phi_{c}^{{}^{\prime }2}\;dV\right)=\frac{\varepsilon_{0}n^{2}}{2}\int 2\phi_{c}^{\prime }\delta \left(\phi_{c}^{\prime }\right)\;dV.$$

We can rearrange this term, if we consider the rules of differentiating a function product

(17) $$\begin{array}{cc} {\left(\phi_{c}\delta \phi_{c}^{\prime }\right)}^{\prime } & =\phi_{c}^{\prime }\delta \phi_{c}^{\prime }+\phi_{c}\delta \phi_{c}^{\prime \prime },\\ \phi_{c}^{\prime }\delta \phi_{c}^{\prime } & ={\left(\phi_{c}\delta \phi_{c}^{\prime }\right)}^{\prime }-\phi_{c}\delta \phi_{c}^{\prime \prime }. \end{array}$$

The integral in Equation (16) can be rewritten as,

(18) $$\begin{array}{cc} \varepsilon_{0}n^{2} & \int \phi_{c}^{\prime }\delta \left(\phi_{c}^{\prime }\right)\;dV=\varepsilon_{0}n^{2}(\underset{=0}{\underset{︸}{\phi_{c}\delta \phi_{c}^{\prime }{|}_{0}^{\infty }}}\;-\\ \; & -\int \phi_{c}\delta \left(\phi_{c}^{\prime \prime }\right)\;dV), \end{array}$$

where the first term on the right-hand side equals 0 at infinity, since we impose the electric potential there to be constant and equal to 0. Taking into account the Poisson’s equation for the water dipoles, namely $\phi_{c}^{\prime \prime }\left(x\right)=\nabla \cdot P/\varepsilon_{0}n^{2}$, where P represents the net polarization of the permanent water dipoles, we get

(19) $$-\varepsilon_{0}n^{2}\int \phi_{c}\delta \left(\phi_{c}^{\prime \prime }\right)\;dV=\int \phi_{c}\delta \rho_{c}\;dV.$$

Here, $\rho_{c}$ corresponds to the bound charge density due to the dipoles’ polarizations, which is related to net polarization $\rho_{c}=-\nabla \cdot P$. We observe that δ(∇·P)=∇·δP. The integral in (Equation (19)) can now be rewritten:

(20) $$\int \phi_{c}\delta \rho_{c}\;dV=-\int \phi_{c}\left(\nabla \cdot \delta P\right)\;dV.$$

The product rule for divergence can be used $\nabla \cdot \left(\phi_{c}\delta P\right)=\left(\nabla \phi_{c}\right)\cdot \delta P+\phi_{c}\left(\nabla \cdot \delta P\right)$, so that the integral of Equation (20) can now be written differently again:

(21) $$\int \phi_{c}\left(\nabla \cdot \delta P\right)\;dV=\underset{=0}{\underset{︸}{\int \nabla \cdot (\phi_{c}\delta P)\;dV}}-\int \left(\nabla \phi_{c}\right)\cdot \delta P\;dV.$$

Here, the first integral on the right hand side vanishes by virtue of the divergence theorem; since the potential far away from the plates is constant and equal to zero. We therefore arrive at the final result

(22) $$\delta F_{1}=\int \left(\nabla \phi_{c}\right)\cdot \delta P\;dV.$$

The polarization, P, is related to the average orientation of all water dipoles (Equation (2)):

(23) $$P\left(x\right)=n_{w}{\langle P\left(x,\omega \right)\rangle }_{\omega }pn_{\phi }.$$

Here, $n_{w}$ is the number density of water molecules in the solution, p=|p| is the internal point-like dipole magnitude, $n_{\phi }=\nabla \phi_{c}/\left|\nabla \phi_{c}\right|$ is the unit vector away from the charged plate and ${\langle P\left(x,\omega \right)\rangle }_{\omega }$ is defined by Equation (15) (see Figure 3). Since our case deals with a negatively charged surface (σ<0), P points in the direction opposite to the direction of the *x*-axis and is thus negative (for details see Reference [76]). Since the variation of P can be written $\delta P\left(x\right)={\langle n_{w}p\delta P\left(x,\omega \right)\rangle }_{\omega }$, we arrive at the variation of $F_{1}$:

(24) $$\delta F_{1}=n_{w}\int {\langle \delta P\left(x,\omega \right)\left(\nabla \phi_{c}\right)\cdot p\rangle }_{\omega }\;dV.$$

Similarly, for $F_{2}$ by taking into account Equation (17), we get

(25) $$\delta \left(\frac{\varepsilon_{0}n^{2}}{2}\int \phi^{{}^{\prime }2}\;dV\right)=\int \phi \delta \rho_{\mathrm{free}}\;dV.$$

The Poisson equation is different for free charges (ions): $\phi^{\prime \prime }\left(x\right)=-\rho_{\mathrm{free}}/\varepsilon_{0}n^{2}=e_{0}\left(n_{+}\left(x\right)-n_{-}\left(x\right)\right)$. The variation by $\phi^{\prime \prime }\left(x\right)$ in Equation (25) can be written with macroscopic net volume charge density $\rho_{\mathrm{free}}\left(x\right)$, which in turn is the sum of the contributions of the local net ion charges. Performing the variation on ion charge distribution $\rho_{\mathrm{free}}\left(x\right)$ gives

(26) $$\delta \rho_{\mathrm{free}}=e_{0}\left(\delta n_{+}-\delta n_{-}\right),$$

finishing the variation of the term $F_{2}$:

(27) $$\delta F_{2}=\int e_{0}\phi \left(\delta n_{+}-\delta n_{-}\right)\;dV.$$

#### 3.1.2. Ion Mixing ($F_{3}$, $F_{4}$ and $F_{5}$)

It makes sense to perform the variation of the ion mixing terms ($F_{3}$ and $F_{4}$), together with their Lagrange multipliers ($F_{5}$), since the variation $\delta n_{+}$ and $\delta n_{-}$ will be a common term for positive and negative ions, respectively. It is easily shown from Equation (14) that

(28) $$\delta F_{3}+\delta F_{4}+\delta F_{5}=kT\int \delta n_{+}\left(\lambda_{+}+\ln\frac{n_{+}}{n_{0}}\right)\;dV+kT\int \delta n_{-}\left(\lambda_{-}+\ln\frac{n_{-}}{n_{0}}\right)\;dV.$$

#### 3.1.3. Dipole Mixing ($F_{6}$ and $F_{7}$)

Variation of the terms $F_{6}$ and $F_{7}$ is straightforward. Since the bulk water number density, $n_{w}$, is taken to be constant, the variation of $F_{6}$ is

(29) $$\delta F_{6}=kTn_{w}\int \left({\langle \delta P\left(x,\omega \right)\ln P(x,\omega )+\delta P\left(x,\omega \right)\rangle }_{\omega }\right)\;dV.$$

The last variation of $F_{7}$ is performed over the probability, P(x,ω), and the Lagrange multiplier, η(x). Expanding and applying the product rule, we find that

(30) $$\delta F_{7}=kTn_{w}\int \left(\delta \eta \left(x\right){\langle P\left(x,\omega \right)\rangle }_{\omega }+\eta \left(x\right){\langle \delta P\left(x,\omega \right)\rangle }_{\omega }-\delta \eta \left(x\right)\right)\;dV.$$

### 3.2. Euler-Lagrange Equations

Combining the variations of all the integrals given in Equation (14), their sum δF gives us the variation of Helmholtz free energy. Factoring all the variation terms with respect to $n_{+}\left(x\right),n_{-}\left(x\right),P\left(x,\omega \right)$ and η(x) gives

(31) $$\begin{array}{cc} \; & \delta F=\int \;dV\delta n_{+}\left(x\right)\left[kT\left(\ln\frac{n_{+}\left(x\right)}{n_{0}}+\lambda_{+}\right)+\phi e_{0}\right]+\int \;dV\delta n_{-}\left(x\right)\left[kT\left(\ln\frac{n_{-}\left(x\right)}{n_{0}}+\lambda_{-}\right)-\phi e_{0}\right]+\\ \; & \;+\int \;dVn_{w}{\langle \delta P\left(x,\omega \right)\left(\nabla \phi_{c}\cdot p+\frac{\ln P(x,\omega )+\eta (x)+1}{\beta }\right)\rangle }_{\omega }+kT\int \;dVn_{w}\delta \eta \left(x\right)\left({\langle P\left(x,\omega \right)\rangle }_{\omega }-1\right). \end{array}$$

The volume differentials in a planar geometry are dV=Sdx. Since the minimization condition demands δF=0, the expressions multiplied by $\delta n_{+}\left(x\right)$, $\delta n_{-}\left(x\right)$, δP(x,ω) and δη(x) in the last equation must equal zero, resulting in a system

(32) $$\begin{array}{c} kT\left(\ln\frac{n_{+}\left(x\right)}{n_{0}}+\lambda_{+}\right)+\phi e_{0}=0, \end{array}$$

(33) $$\begin{array}{c} kT\left(\ln\frac{n_{-}\left(x\right)}{n_{0}}+\lambda_{-}\right)-\phi e_{0}=0, \end{array}$$

(34) $$\begin{array}{c} E_{c}p\cos\omega +\frac{\ln P(x,\omega )+\eta (x)+1}{\beta }=0, \end{array}$$

(35) $$\begin{array}{c} {\langle P\left(x,\omega \right)\rangle }_{\omega }-1=0. \end{array}$$

Here, we write β=1/kT and expand the dot product $\nabla \phi_{c}\cdot p=E_{c}p\cos\omega$ (see Figure 3). Solving Equations (32) and (33), we obtain the ion spatial distributions

(36) $$n_{+}\left(x\right)=n_{0}\exp\left(-\beta e_{0}\phi -\lambda_{+}\right),$$

(37) $$n_{-}\left(x\right)=n_{0}\exp\left(\beta e_{0}\phi -\lambda_{-}\right).$$

The boundary conditions state that ϕ(x→∞)=0 and $n_{+,-}\left(x\to \infty \right)=n_{0}$, which renders $\lambda_{+}=\lambda_{-}=0$. We may now turn our attention to the variation of permanent water dipoles orientation. Solving for P(x,ω), Equation (34) gives

(38) $$P\left(x,\omega \right)=\Lambda \left(x\right)\exp\left(-\beta E_{c}p\cos\omega \right),$$

where Λ(x)=exp(−η(x)−1). Substituting the cavity field $E_{c}$ by *E* (Equation (4)) and dipole moment magnitude *p* by $p_{0}$ (Equation (5)) gives

(39) $$P\left(x,\omega \right)=\Lambda \left(x\right)\exp\left(-\beta \frac{3E}{2}\left(\frac{2+n^{2}}{3}\right)p_{0}\cos\omega \right),$$

where $p_{0}$ is the magnitude of $p_{e}$. The final result is expressed using the constant γ defined in Equation (9):

(40) $$P\left(x,\omega \right)=\Lambda \left(x\right)\exp\left(-\beta \gamma Ep_{0}\cos\omega \right).$$

We can now evaluate the average internal dipole moment by integrating over mean orientations (considering Equation (23)),

(41) $$\begin{array}{ccc} \; & p\langle \cos\omega \rangle =p_{0}\left(\frac{2+n^{2}}{3}\right)\langle \cos\omega \rangle \\ \; & =\frac{\int_{0}^{\pi }\left(\frac{2+n^{2}}{3}\right)p_{0}\cos\omega \exp\left(-\beta \gamma Ep_{0}\cos\omega \right)\;d\Omega }{\int_{0}^{\pi }\exp(-\beta \gamma Ep_{0}\cos\omega )\;d\Omega } & \\ \; & =-p_{0}\left(\frac{2+n^{2}}{3}\right)L\left(\beta \gamma Ep_{0}\right). \end{array}$$

The orientational polarization of water is thus (see Equations (2) and (5)):

(42) $$\begin{array}{ccc} P(x) & = & n_{w}p\langle \cos\omega \rangle \\ & = & -n_{w}p_{0}\left(\frac{2+n^{2}}{3}\right)L\left(\beta \gamma E\left(x\right)p_{0}\right). \end{array}$$

If we insert the above result and the ion distribution functions (Equations (36) and (37)) into the average microscopic charge density equation $\rho \left(x\right)=\rho_{\mathrm{free}}\left(x\right)-dP/dx$ [61,77], where $\rho_{\mathrm{free}}$ is the contribution of the net ion charges Equations (26), (36) and (37) and P(x) is the polarization due to partially oriented water dipoles, we get the expression for ρ(x) in a one-dimensional case:

(43) $$\rho \left(x\right)=-2e_{0}n_{0}\sinh\left(\beta e_{0}\phi \left(x\right)\right)+n_{w}p_{0}\left(\frac{2+n^{2}}{3}\right)\frac{d}{dx}\left(L\left(\beta \gamma E\left(x\right)p_{0}\right)\right).$$

Inserting the above expression for average microscopic volume charge density ρ(x) into the Poisson’s equation,

(44) $$\phi^{\prime \prime }\left(x\right)=-\frac{\rho (x)}{n^{2}\varepsilon_{0}},$$

we get the modified LPB differential equation for the electric potential ϕ(x):

(45) $$\phi^{\prime \prime }\left(x\right)=\frac{1}{n^{2}\varepsilon_{0}}\left[2e_{0}n_{0}\sinh(\beta e_{0}\phi \left(x\right))-n_{w}p_{0}\left(\frac{2+n^{2}}{3}\right)\frac{d}{dx}\left(L\left(\beta \gamma E\left(x\right)p_{0}\right)\right)\right],$$

where $\phi^{\prime \prime }\left(x\right)$ is the second derivative of the electric potential ϕ(x) with respect to *x* and $E\left(x\right)=-\phi^{\prime }\left(x\right)$. Equation (45) can be factorized via a product rule if we take into account that the Langevin function is odd and its derivative is $L^{\prime }\left(u\right)=1/u^{2}-1/\sinh^{2}u$ in the following form [7]:

(46) $$\frac{d}{dx}\left[\varepsilon_{0}\varepsilon_{r}\left(x\right)\phi^{\prime }\left(x\right)\right]=2e_{0}n_{0}\sinh\left(\beta e_{0}\phi \left(x\right)\right),$$

(47) $$\varepsilon_{r}\left(x\right)=n^{2}+n_{w}\frac{p_{0}}{\varepsilon_{0}}\left(\frac{2+n^{2}}{3}\right)\frac{L(\beta \gamma E\left(x\right)p_{0})}{E(x)},$$

where $\varepsilon_{r}\left(x\right)$ is the relative permittivity (Equation (11)). This modified Langevin Poisson-Boltzmann (LPB) differential equation (Equation (46)) is subject to two boundary conditions. The first boundary condition arises from the electro-neutrality of the system, which assumes that the total net charge of the system is zero, hence

(48) $$\int \rho_{\mathrm{free}}\left(x\right)\;dV-\sigma S=0,$$

where σ is the negative membrane surface charge density, *S* is the total membrane surface area and $\rho_{\mathrm{free}}\left(x\right)=-2e_{0}n_{0}\sinh\left(\beta e_{0}\phi \left(x\right)\right)$ is the macroscopic (net) volume charge density of coions and counterions. Since the macroscopic volume charge density is only dependent on *x* (Equation (43)) and the differential dV=Sdx, Equation (48) may be rewritten

(49) $$\int_{0}^{\infty }2e_{0}n_{0}\sinh\left(\beta e_{0}\phi \left(x\right)\right)\;dx=-\sigma .$$

If we integrate Equation (45) once over the whole system, we get

(50) $$\phi^{\prime }\left(x=0\right)=-\frac{1}{n^{2}\varepsilon_{0}}\left[\sigma +n_{w}p_{0}\left(\frac{2+n^{2}}{3}\right)\cdot L\left(\beta \gamma Ep_{0}{|}_{x=0}\right)\right].$$

The second boundary condition states that the electric potential far away from the charged surface (in the bulk) is constant $\phi^{\prime }\left(x\to \infty \right)=0$, rendering $L(\beta \gamma Ep_{0}{|}_{x\to \infty })=0$. The modified LPB equation (Equation (46)) was derived in one dimension, but can be rewritten in a more general form to apply to an arbitrary three-dimensional geometry. In three dimensions, the steps are analogous and discussed in detail in a previous work [58], where a three-dimensional Lagrangian was derived for a model of finite-sized ions. With this in mind, the modified LPB equation (Equation (46)) can be rewritten:

(51) $$\nabla \cdot \left[\varepsilon_{0}n^{2}\nabla \phi \left(r\right)\right]+n_{w}p_{0}\left(\frac{2+n^{2}}{3}\right)\nabla \cdot \left(n_{\phi }\;L\left(\beta \gamma Ep_{0}\right)\right)=2e_{0}n_{0}\sinh(\beta e_{0}\phi \left(r\right)),$$

where $n_{\phi }=\nabla \phi /\left|\nabla \phi \right|=\nabla \phi /E$. We may factor the last equation, so that

(52) $$\nabla \cdot \left[\varepsilon_{0}\left(n^{2}+\frac{n_{w}p_{0}}{\varepsilon_{0}}\left(\frac{2+n^{2}}{3}\right)\frac{L\left(\beta \gamma Ep_{0}\right)}{E}\right)\nabla \phi \left(r\right)\right]=2e_{0}n_{0}\sinh(\beta e_{0}\phi \left(r\right)).$$

This modified LPB equation can be written even more compactly, considering the definition of spatially dependent permittivity $\varepsilon_{r}\left(r\right)$ given by Equation (47) (for details, see Reference [58]):

(53) $$\nabla \cdot \left[\varepsilon_{0}\varepsilon_{r}\left(r\right)\nabla \phi \left(r\right)\right]=2e_{0}n_{0}\sinh\left(\beta e_{0}\phi \left(r\right)\right),$$

(54) $$\varepsilon_{r}\left(r\right)=n^{2}+n_{w}\frac{p_{0}}{\varepsilon_{0}}\left(\frac{2+n^{2}}{3}\right)\frac{L(\beta \gamma E\left(r\right)p_{0})}{E(r)}.$$

Here, $\rho_{\mathrm{free}}\left(r\right)$ is the macroscopic (net) volume charge density of coions and counterions. A corresponding three-dimensional variant of the boundary condition (Equation (50)) is

(55) $$\nabla \phi \left(r=r_{\mathrm{surf}}\right)=-\frac{1}{n^{2}\varepsilon_{0}}\left[\sigma n_{\phi }+n_{\phi }n_{w}p_{0}\left(\frac{2+n^{2}}{3}\right)\cdot L\left(\beta \gamma E\left(r\right)p_{0}\left(r\right){|}_{r=\mathrm{surf}}\right)\right].$$

Rearranging, it follows that

(56) $$\nabla \phi \left(r=r_{\mathrm{surf}}\right)\left[1+\frac{n_{\phi }}{\nabla \phi (r=r_{\mathrm{surf}})}\frac{n_{w}p_{0}}{n^{2}\varepsilon_{0}}\left(\frac{2+n^{2}}{3}\right)\cdot L\left(\beta \gamma E\left(r\right)p_{0}\left(r\right){|}_{r=\mathrm{surf}}\right)\right]=-\frac{\sigma }{n^{2}\varepsilon_{0}}n_{\phi }.$$

Evaluating the second expression on the left hand side of the last equation gives

(57) $$\frac{n_{\phi }}{\nabla \phi (r=r_{\mathrm{surf}})}=\frac{\nabla \phi (r=r_{\mathrm{surf}})}{\left|\nabla \phi (r=r_{\mathrm{surf}})\right|}\frac{1}{\nabla \phi (r=r_{\mathrm{surf}})}=\frac{1}{E(r=r_{\mathrm{surf}})}.$$

Combining this simplification with Equation (42), Equation (55) becomes

(58) $$\nabla \phi \left(r=r_{\mathrm{surf}}\right)\varepsilon_{r}\left(r=r_{\mathrm{surf}}\right)=-\frac{\sigma n_{\phi }}{\varepsilon_{0}}.$$

Here we also take into account the expression for $\varepsilon_{r}$ (Equation (47)). We see that the term inside the square brackets on the left-hand side of Equation (56) is precisely the definition of the relative permittivity on the surface of charged membrane $\varepsilon_{r}\left(r=r_{\mathrm{surf}}\right)$ (Equation (54)), yielding the general result

(59) $$\nabla \phi \left(r=r_{\mathrm{surf}}\right)=-\frac{\sigma n_{\phi }}{\varepsilon_{0}\varepsilon_{r}\left(r=r_{\mathrm{surf}}\right)}.$$

## 4. Results

**Figure 4:**
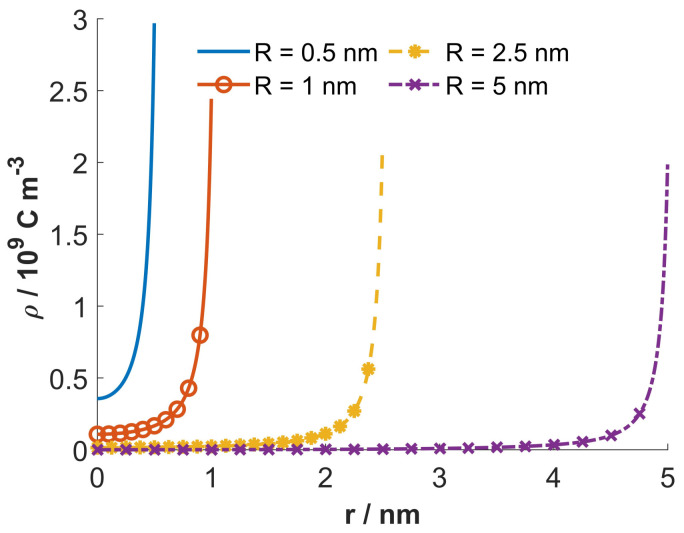
Macroscopic (net) volume charge density of coions and counterions ($\rho_{\mathrm{free}}$) as a function of the radial distance from the geometrical axis of tube (*r*) calculated for 4 values of the inner tube diameter *R*: 0.5 nm, 1.0 nm, 2.5 nm and 5.0 nm. The bulk concentrations of counterions and coions $n_{0}/N_{A}=0.15\;\mathrm{mol}/L$ and $\sigma =-0.25\;\mathrm{As}/m^{2}$, T=298K, constant concentration of water $n_{w}/N_{A}=55\;\mathrm{mol}/L$, optical refractive index n=1.33 and magnitude of external dipole moment of water $p_{0}=3.1\;\mathrm{Debye}$, where $N_{A}$ is the Avogadro number.

**Figure 5:**
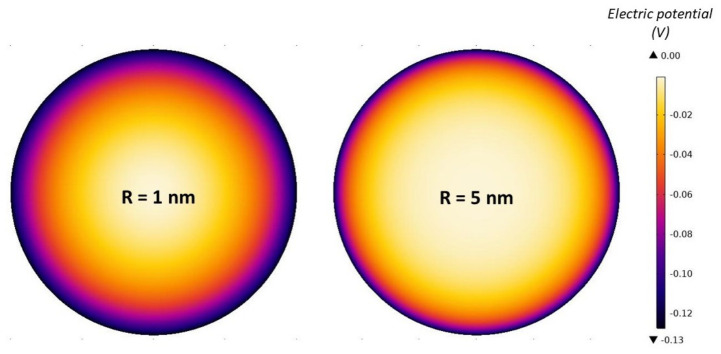
Space dependence of electric potential in the cross-section of the tube interior calculated for 2 values of the inner tube diameter *R*: 1.0 nm and 5.0 nm. The values of the model parameters are the same as given at Figure 4.

Figure 4 shows the dependency of the calculated macroscopic (net) volume charge density of the electrolyte solution inside the nanotubes ($\rho_{\mathrm{free}}\left(r\right)$) on the radial distance from the geometrical axis of the tube. It can be seen in the figure that for larger radii of the inner cross-sections of the nanotubes (*R*), the value $\rho_{\mathrm{free}}$ at the geometrical axis of the tube is zero, which means that the number densities of counterions and coions there are equal and the electric potential is constant, that is, zero in our case (see the right panel in Figure 5).

**Figure 6:**
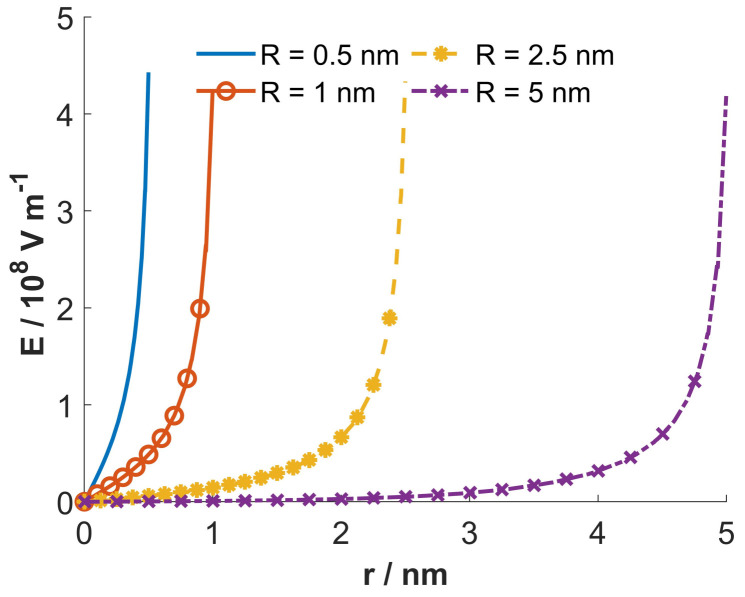
The magnitude of electric field strength as a function of the radial distance from the geometrical axis of tube (*r*), calculated for 4 values of the inner tube diameter *R*: 0.5 nm, 1.0 nm, 2.5 nm and 5.0 nm. The values of the model parameters are the same as given at Figure 4. The narrow nanotube before and after entrance of the nanoparticles.

On the contrary, for smaller values of the nanotube radius *R*, the value of $\rho_{\mathrm{free}}$ at geometrical axis of the nanotube is not zero (Figure 4). Accordingly, for small values of the radius of the inner nanotube also the gradient of the electric field (Figure 6) and the electric potential at the nanotube geometrical axis are not zero (left panel in Figure 5). Hence, the bulk condition of the equal number densities of counterions and coions is fulfilled outside the interior of the nanotube.

**Figure 7:**
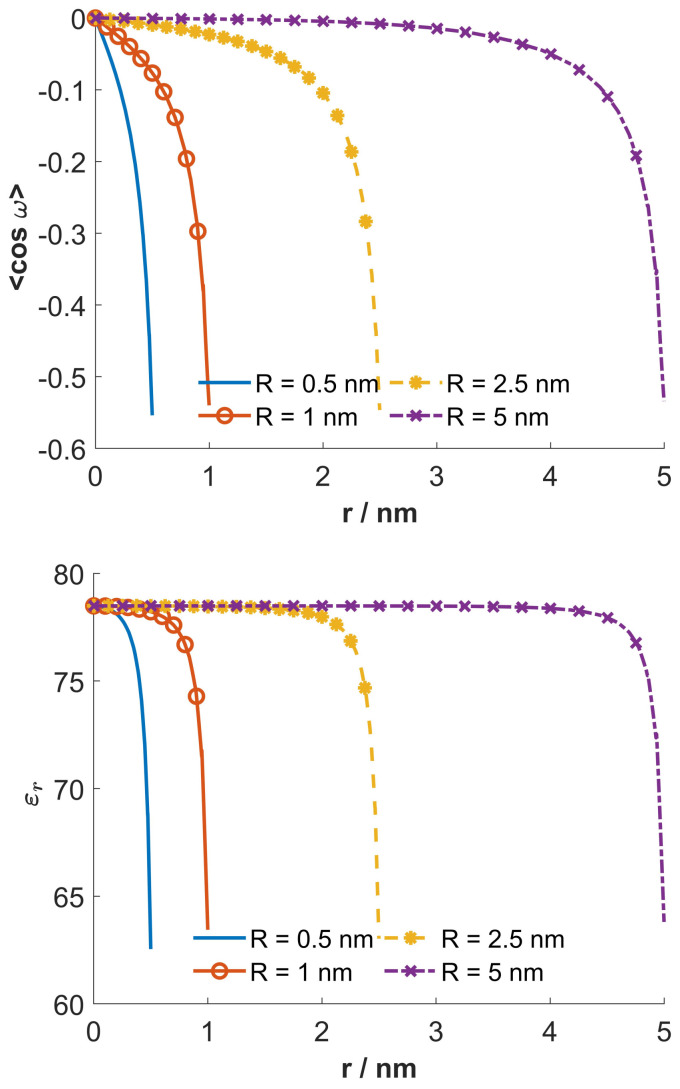
Average orientation ${\langle \cos\left(\omega \right)\rangle }_{\omega }$ and relative permittivity $\varepsilon_{r}$ as a function of the radial distance from the geometrical axis of tube (*r*), calculated for 4 values of the inner tube diameter *R*: 0.5 nm, 1.0 nm, 2.5 nm and 5.0 nm. The values of the model parameters are the same as given at Figure 4.

Figure 7 shows the dependency of the average orientation ${\langle \cos\left(\omega \right)\rangle }_{\omega }$ and the relative permittivity $\varepsilon_{r}$ on the radial distance from the geometrical axis of tube (*r*), calculated for four different values of nanotube inner radius *R*. It can be seen that for small radii, *R*, the average orientational of water dipoles is relatively strong also in the vicinity of geometrical axis of the tube, while for larger R the average orientation of water dipoles is strong only in the region near the charged inner surface of the tube.

## 5. Discussion and Conclusions

In this paper, we derived a modified Langevin Poisson-Boltzmann (LPB) model and the modified LPB equation to theoretically describe the electric double layer (EDL) for a monovalent electrolyte solution inside very narrow nanotubes with a negatively charged inner surface. In the modified LPB approach, the electronic polarization of the water is taken into account by assuming a permanent dipole embedded in the center of the sphere with a volume equal to the average volume of a water molecule. The effect of a polarizing environment is reproduced by introduction of the cavity field in the saturation regime [7,61,76]. In past EDL studies, treatments of cavity fields and structural correlations between water dipoles were limited to cases of relatively small electric field strengths, far away from the saturation limit of polarization and orientational ordering of water molecules [73,74,75]. High magnitudes of electric field strength were later added in several works [44,61,63,78]. A commonly oversimplified assumption when theoretically describing the EDL is the assumption of a surface charge density-independent relative permittivity in the inner (Stern) layer. Due to orientational ordering of water dipoles, the relative permittivity of the Stern layer depends on the electric field strength, that is, on the surface charge density (σ) of the electrode [51,79,80,81,82]. Furthermore, fitting the model curves with a range of free parameters to the experimental points [83] cannot prove that the Stern layer capacitance and permittivity is σ-independent. The decrease in the relative permittivity close to the charged surface (electrode) is obviously partially the consequence of orientational ordering of water dipoles close to the saturation regime or in the saturation regime as shown theoretically in References [6,27,44,54,58,59,61,62,63,64,80,82].

Within a recently presented phenomenological approach it is claimed that close to the charged surface, almost all water molecules belong to water shells around the ions, while the free water molecules are excluded [83]. The results of simulations clearly refute this fact [84] by showing increased water ordering in the direction towards the charged surface (including the region close to the charged surface) (Figure 7, upper panel) even for high salt concentrations [84], in quantitative agreement with mean-field theoretical predictions [7,82]. For example, for a magnitude of $0.16\;{\mathrm{As}/m}^{2}$ surface charge density, there is practically no difference in the orientational ordering and space distribution of water dipoles close to the charged surface between water with and without NaCl (of concentration 500 mmol/L) [84]. In general, for magnitudes of surface charge density up to around $0.3\;\mathrm{As}/m^{2}$, where the mean-field approach can still be justified [7,82], there is only a weak quantitative influence of salt on the profile of orientational ordering of water dipoles in Stern and diffuse layers, but not qualitative [84]. Note that the multi-layering of water predicted in simulations [84] cannot be predicted within our mean-field approach [44,61] as well also not in the oversimplified phenomenological models [83].

Besides the saturation in polarization/water dipole orientation at high magnitudes of the electric field strength, the important thing to consider in the EDL studies is also the saturation in the counterion concentration near the charged surface due to the finite size of ions. These steric effects were first predicted in the Wicke-Eigen’s model (also called the Bikerman’s model) and their modifications [3,5,22,25,27,35,85,86]. For finite sized ions, the dielectric permittivity profile in the vicinity of a charged surface is modulated by the depletion of water dipoles at the charged surface due to accumulated counterions [58,82]. In the modified LPB model [7,59], described in the present paper, these steric effects were not taken into account.

The described decrease in the relative permittivity relative to its bulk value in the present paper is the consequence of strong orientational ordering of the water dipoles in the vicinity of the charged surface (Figure 7). Contrary to our results it is claimed in Reference [87] that the relative permittivity is increased in direction to the charged surface. As pointed out in publications of different authors the predicted increase of relative permittivity near the charged surface in Reference [87] is unphysical [6,59] and defies the common wisdom in electrochemistry [56]. In addition, the experiments report just the converse as predicted in Reference [87], that is, the experiments indicated the decrease of relative permittivity near the charged surface [88,89]. The predicted substantial increase of relative permittivity in the inner part of the double layer near the charged surface in Reference [87] is due to arise in the dipole density near the surface as pointed out in Reference [56]. This unphysical result [6] is the consequence of inconsistency of so-called dipolar PB theory presented in Reference [87] as indicated in Reference [59]. Namely, it was shown [59] that the dipolar PB theory for point-like ions in Reference [87] assumes an orientationally averaged Boltzmann factor for spatial distribution function for water dipoles, which is however not compatible with the assumption of point-like ions. Energy dependent spatial distribution of water dipoles cannot be taken into account simultaneously with the assumption of point-like ions, but only if the finite size of molecules in the electrolyte solution are taken into account [35,61]. This means that the dipolar PB model presented in Reference [87] is not a self-consistent model and consequently predicts unphysical results which are not compatible with experimental results even qualitatively, as noticed in References [6,56,59] and other publications.

The other important difference between our modified LPB model and the theoretical model presented in Reference [87] is that our value for (external) water dipole moment 3.1 D [7,44,51,61] is considerably smaller than the corresponding value 4.86 D used in Reference [87]. The value 3.1 D is closer to the experimental values of the effective dipole moment of water molecules in clusters (2.7 D) and in bulk solution (2.4–2.6 D) (see for example Reference [68]). The value 4.86 D is so large in order to compensate for the cavity field [6,61,74,75,78] that is not taken into account in Reference [87], as noticed also in Reference [6], but is considered in the present modified LPB model. The model value 3.1 D can be additionally decreased by taking into account structural correlations between water dipoles [60,78]. The ion-ion and ion-water correlations were taken into account also in the mean-field models of References [8,65,66].

It has been shown that for finite-sized ions the drop in the number density of water near a charged surface results in an additional decrease of permittivity [7,58]. A further generalization of the modified LPB model with steric effects taken into account within a lattice-statistics model of a modified LPB is found in References [44,51,82]. By taking into account asymmetric finite size of ions the modified LPB equation was generalized to (modified Langevin Eigen-Wicke model) [44,51,82]:

(60) $$\frac{d}{dx}\left[\varepsilon_{0}\varepsilon_{r}\left(x\right)\frac{d\phi }{dx}\right]=2e_{0}n_{s}n_{0}\frac{\sinh\left(\beta e_{0}\phi \right)}{D_{A}\left(\phi ,E\right)},$$

where $\varepsilon_{r}\left(x\right)$ is the spatial dependence of relative permittivity:

(61) $$\varepsilon_{r}\left(x\right)=n^{2}+n_{0w}n_{s}\frac{p_{0}}{\varepsilon_{0}}\left(\frac{2+n^{2}}{3}\right)\left(\frac{F(\gamma p_{0}E\beta )}{D_{A}\left(\phi ,E\right)E}\right)$$

and

(62) $$D_{A}\left(\phi \right)=\alpha_{+}n_{0}e^{-e_{0}\phi \beta }+\alpha_{-}n_{0}e^{+e_{0}\phi \beta }+\frac{n_{0w}}{\gamma p_{0}E\beta }\sinh\left(\gamma p_{0}E\beta \right).$$

Here, the parameters $\alpha_{+}$ and $\alpha_{-}$ are the number of lattice sites occupied by a single positive and negative hydrated ion, respectively, where a single water molecule is assumed to occupy just one lattice site. The reduced number density of lattice sites $n_{s}/N_{A}=55\;\;\mathrm{mol}/L$ is equal to the concentration of pure water [44,51,82]. The symbol $n_{0w}$ stands for the bulk number density of water molecules. The function F(u) is defined as F(u)=L(u)sinh(u)/u, where L(u) is the Langevin function.

The results of the present paper are important when considering electric fields within artificial as well as biological channels containing an electrolyte. Much attention has recently been given to understanding tunneling nanotubes (TNTs), small tubular structures that drive cell communication and spreading of pathogens [12]. Not yet fully understood, it is thought that these tubular structures initiate from local membrane bending facilitated by laterally distributed proteins or anisotropic membrane nanodomains. Further research is needed to clarify the role of EDL in the inception of these structures, since cytoplasmatic proteins and other elements are electrically charged. When such motor proteins are complemented by protruding cytoskeletal forces provided by the polymerization of f-actin, TNT formation is crucial in determining cell morphology, sometimes even leading to endovesiculation of the red blood cell membrane [90,91,92]. Recently, within a molecular mean-field approach and taking into account the asymmetric size of ions, polarization of water, and ion-ion and ion-water correlations, the ionic and water flows through biological ion channels was theoretically considered [65,66].

To conclude, in the present paper we started from a mean-field Helmholtz free energy functional, presented a thorough derivation of the modified LPB equation and model by minimization of the system free energy for the case of planar geometry. A special emphasis was devoted to orientational ordering of water dipoles, taken into account in the expression for the free energy by rotational entropy. Our approach provides a distinct analytical description of the interplay between mean-field electrostatic and entropic effects arising from the mixing entropy of ions and rotational entropy of water dipoles in EDL.

The derived modified LPB equation in planar geometry is then generalized for arbitrary geometry and then used to calculate numerically the average orientation of water dipoles, relative permittivity $\varepsilon_{r}$, magnitude of electric field strength, electric potential and the macroscopic (net) volume charge density of coions and counterions for a cylindrical geometry (in dependence on radial distance from the center of the tube).

Among other things it is indicated that in the saturation regime close to the charged surface, where the magnitude of electric field is very large (Figure 6), strong orientational water dipole ordering (Figure 7, upper panel) may result in a strong local decrease of permittivity (Figure 7, lower panel). The relative permittivity of the electrolyte solution decreases with increasing magnitude of the electric field strength.

**Figure 8:**
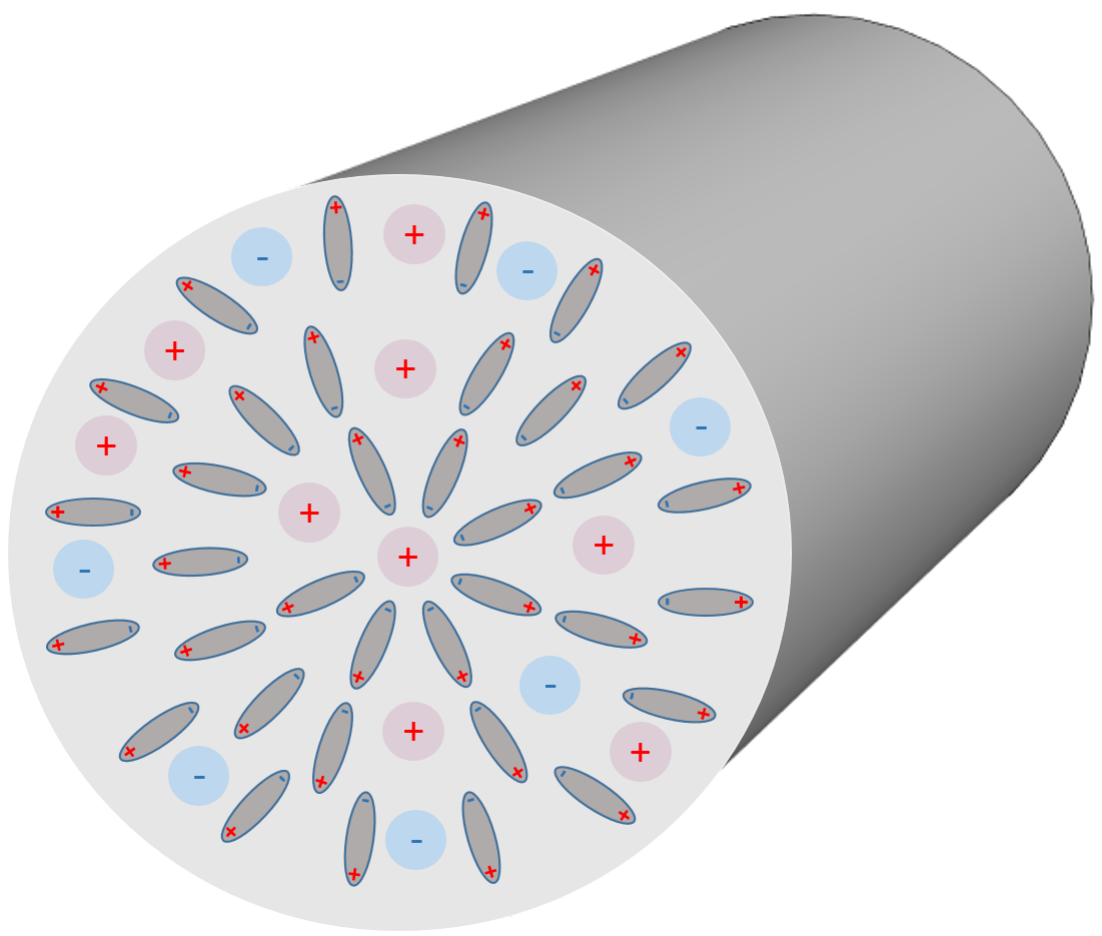
A schematic figure of a radial arrangement of water dipoles inside a very narrow cylindrical nanotube. The inner surface of the tube is negatively charged.

Most interesting, we have shown that in the case of very narrow nanotubes the macroscopic (net) volume charge density of coions and counterions ($\rho_{\mathrm{free}}$) at geometrical axis of the nanotube is not zero (Figure 4). In addition, in narrow nanotubes the water dipoles are partially oriented also close to the axis of the nanotube (Figure 7, upper panel), as schematically shown in (Figure 8). The potential importance of this phenomena for the transport through the narrow channels with the charged inner surface, specific only for very narrow nanotubes, should be investigated in the future. The channels in biological membranes can be an interesting example of such systems.

## 6. Materials and Methods

To solve Equation (52), a partial differential equation, we have used Comsol Multiphysics and its electrostatics stationary solver. The mesh consists of 4946 elements, the boundary condition (Equation (59)) was applied on the 2D cross-section of the nanotube and the geometry was solved for 10293 DoFs. The numerical results were solved using a stationary nonlinear solver (Automatic (Newton)), which implements a damped Newton’s approach, with a minimum damping factor of $10^{-6}$.

## Abbreviations

The following abbreviations are used in this manuscript:

EDL	Electric double layer
modified LPB	modified Langevin Poisson-Boltzmann
